# A rare subpopulation of melanoma cells with low expression of metastasis suppressor NME1 is highly metastatic *in vivo*

**DOI:** 10.1038/s41598-020-58996-3

**Published:** 2020-02-06

**Authors:** Devin Snyder, Ying Wang, David M. Kaetzel

**Affiliations:** 1 Department of Biochemistry and Molecular Biology, School of Medicine, University of Maryland-Baltimore, Baltimore, MD USA; 2 Marlene and Stewart Greenebaum Comprehensive Cancer Center, School of Medicine, University of Maryland-Baltimore, Baltimore, MD USA

**Keywords:** Metastasis, Melanoma

## Abstract

Despite recent advances in melanoma treatment, metastasis and resistance to therapy remain serious clinical challenges. NME1 is a metastasis suppressor, a class of proteins which inhibits metastatic spread of cancer cells without impact on growth of the primary tumor. We have identified a rare subpopulation of cells with markedly reduced expression of NME1 (NME1^*LOW*^) in human melanoma cell lines. To enable isolation of viable NME1^*LOW*^ cells for phenotypic analysis by fluorescence-activated cell sorting (FACS), a CRISPR-Cas9-mediated approach was used to attach an EGFP coding module to the C-terminus of the endogenous NME1 gene in melanoma cell lines. NME1^*LOW*^ cells displayed enhanced collective invasion *in vitro* when implanted as 3D aggregates in Matrigel. NME1^*LOW*^ cells were also highly metastatic to lung and liver when xenografted subcutaneously in immune-deficient NSG mice. RNA-seq analysis revealed that NME1^*LOW*^ cells express elevated levels of genes associated with tumor aggressiveness, as well as with morphogenesis of tissues of neural crest-like origin (melanocytes and neurons, bone and heart tissues; GO: 0009653). The highly malignant NME1^*LOW*^ variant of melanoma cells has potential to provide novel therapeutic targets and molecular markers for improved clinical management of patients with advanced melanoma.

## Introduction

Human tumors are comprised of a diverse network of cells, with specific cell populations primed for enhanced tumor initiation, invasion, and metastasis^1–3^. Most cancer therapies are designed to target primary tumors as composites of phenotypically similar cells that are equally susceptible to treatment^4^. However, genetic and phenotypic aberrations present in rare subpopulations of primary tumor cells can permit survival in the presence of treatment, leading to tumor recurrence, progression and metastasis. Heritable variation, also termed clonal evolution, occurs through an accumulation of genomic mutations during tumor cell proliferation and progression^3,5,6^. Mathematical modeling of clonal evolution in primary and metastatic tumors indicates that most metastases are driven by the same mutational profile as the primary tumor of origin^7^. Heterogeneity within tumor cell populations also occurs in a non-heritable form through alterations in the epigenome, transcriptome and proteome^2,3,5,6^, and this has been suggested to play a more dominant role in promoting metastatic potential^5^.

Metastasis suppressor proteins inhibit metastatic activity with little or no impact on initiation or growth of tumors^8^. The first metastasis suppressor protein to be identified, NME1, was characterized by virtue of its low expression of its mRNA in metastatic melanoma cell lines relative to poorly metastatic counterparts^9^. Subsequent studies have demonstrated involvement of NME1 in regulation of cytoskeletal rearrangements, as well as transcriptional regulation and DNA repair processes^10–13^. While multiple studies have reported an association between reduced NME1 expression and more aggressive forms of melanoma in human patients^14^, others observed little or no correlation^15,16^. As these measurements of NME1 expression were conducted in whole tumor specimens, they did not address the possible existence of rare subpopulations of NME1-deficient cells which could well possess enhanced metastatic properties and represent a stronger index of melanoma progression. To this end, we recently demonstrated the presence of a rare subpopulation with nearly undetectable levels of NME1 expression in spheroid cultures derived from different melanoma cell lines^17^. In the current study, we demonstrate that this subpopulation of melanoma cells (NME1^*LOW*^) exhibits a unique transcriptomic profile characteristic of a neural crest-like phenotype and is highly metastatic when xenografted in immunocompromised NSG mice. Our study suggests the possible existence of NME1^*LOW*^ cells in melanoma tumors that possess enhanced potential for tumor progression and metastatic activity.

## Results

### Melanoma cell lines contain a rare population of cells with low NME1 expression

**Figure 1:**
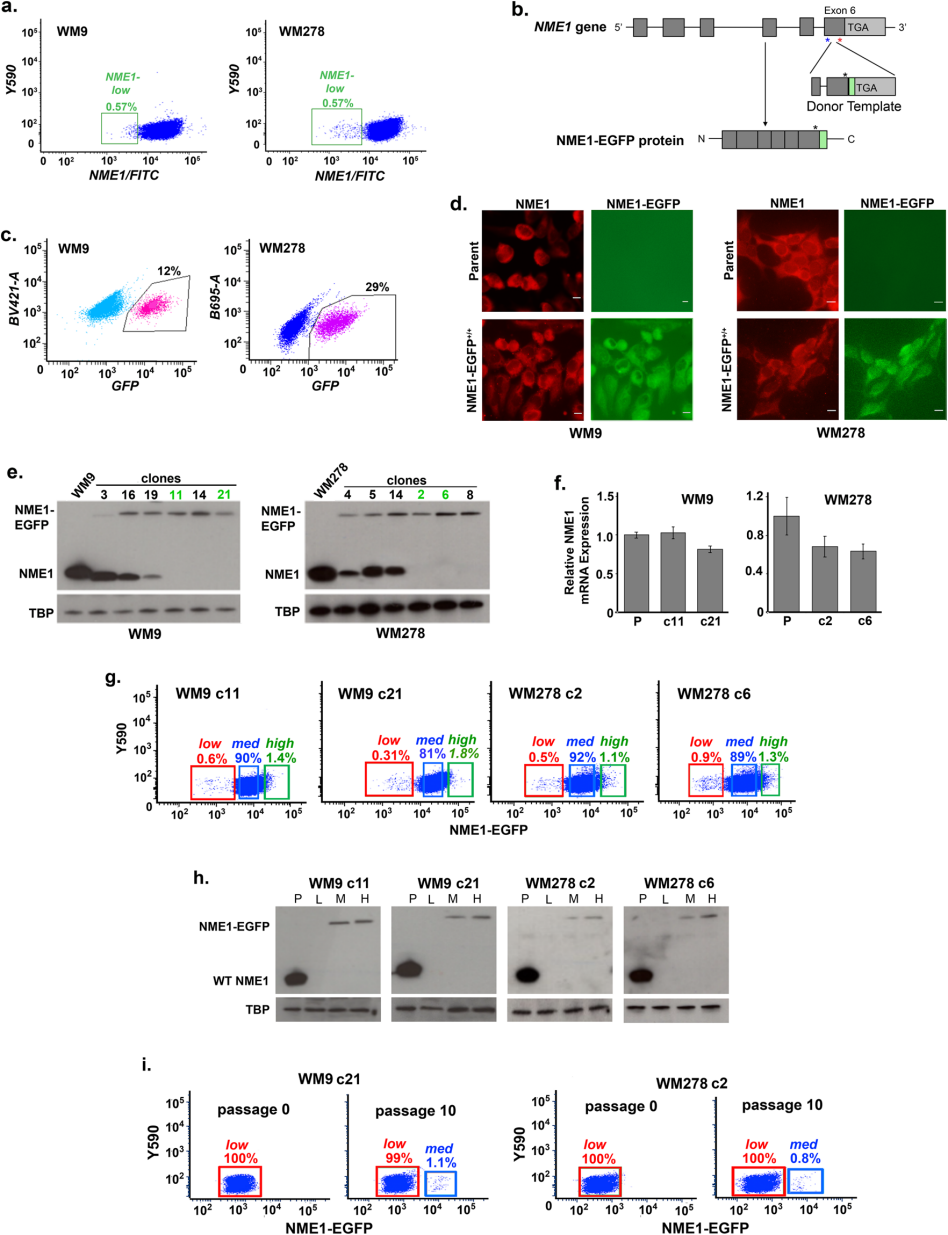
Melanoma cell lines harbor a rare subpopulation that expresses low amounts of the metastasis suppressor NME1. (**a**) Human melanoma cell lines WM9 and WM278 were subjected to immunofluorescent staining for intracellular NME1 and analyzed by flow cytometry. Green boxes highlight subpopulations that express low amounts of NME1. (**b**) Schematic representation of CRISPR/Cas9 (double nickase)-mediated insertion of DNA sequence encoding an EGFP fluorescence-generating peptide tag at the C-terminal coding sequence of the genomic *NME1* locus. Blue and red asterisks indicate recognition sites for sgRNA1 and sgRNA2, respectively. A synonymous mutation is identified with a black asterisk. (**c**) FACS of EGFP-positive cells following electroporation of WM9 and WM278 cell lines with Cas9, sgRNAs and donor template. (**d**) Addition of the C-terminal EGFP tag does not alter the predominantly cytoplasmic staining pattern of wild-type NME1 protein. EGFP-positive cells from WM9 and WM278 lines in panel c were isolated by FACS and examined by fluorescent microscopy after staining with anti-NME1 antibody or imaging for EGFP fluorescence. (**e**) Immunoblot analysis of wild-type NME1 and NME1-EGFP fusion proteins in WM9 and WM278 clones derived from CRISPR/Cas9-mediated recombination. Mobilities of wild-type NME1 and the NME1-EGFP fusion protein (upper blots) and TATA-binding protein (TBP, lower panels) are identified. (**f**) Addition of the C-terminal EGFP tag does not alter expression of the cognate transcript in WM9- and WM278-derived clones. (**g**) NME1-EGFP-expressing clones exhibit the same profile of cellular heterogeneity in NME1 expression seen with the wild-type protein. Subpopulations were divided as shown into three categories based on their expression of EGFP: low (red boxes), medium (blue boxes) and high (green boxes). (**h**) Immunoblot analysis of NME1-EGFP expression in clones derived from the WM9 (clones 11 and 21) and WM278 (clones 2 and 8) cell lines. (**i**) Subpopulations from WM9 and WM278 clones that express low levels of NME1-EGFP retain their low expression phenotype after extensive passaging (10 passages) in culture. Original non-cropped images of the scanned immunoblot membranes in panels (a) and (h) are shown in Figs S3a and b, respectively.

Melanoma cell lines and tumors are composed of subpopulations with distinct profiles of gene expression patterns that impact their initiation, invasion and metastatic activities^17–20^. Some studies have identified cell subpopulations that exhibit distinct differences in their ability to initiate formation of tumor spheres in non-adherent cell culture conditions^17,18^. Melanoma cell subpopulations found under monolayer cell culture conditions also exhibit differences in sphere formation and tumor-initiating activity *in vivo*^20^. Having recently observed that spheroids derived from melanoma cell lines exhibit cellular heterogeneity in expression of the metastasis suppressor NME1^17^, we investigated the expression pattern of NME1 under monolayer culture conditions. A small subpopulation of cells was identified that expressed low amounts of NME1 protein (NME1^*LOW*^; Fig. 1a) in cell lines derived from both metastatic (WM9) and vertical growth phase (WM278) melanomas.

### CRISPR/Cas9-mediated generation of melanoma cell lines that express the fusion protein NME1-EGFP

To isolate viable subpopulations of cells for functional characterization based on their level of NME1 expression, CRISPR/Cas9 technology was used to insert an EGFP-encoding DNA sequence in direct fusion with the C-terminal coding sequence of the genomic *NME1* locus (Fig. 1b). The encoded NME1-EGFP fusion protein (~47 kDa) would enable fluorescence-activated cell sorting (FACS) to capture viable cell subpopulations based on their expression of NME1^21^. Importantly, expression of NME1-EGFP would be controlled by the endogenous *NME1* promoter, thereby maintaining the naturally-occurring profile of heterogeneous NME1 expression.

The EGFP cassette was inserted into the *NME1* gene using the CRISPR-Cas9 Double Nickase System, which relies on mutated Cas9 (Cas9D10A) and two sgRNAs to minimize off-target effects^22^. Predictive software (CHOPCHOP)^23^ indicated that a single sgRNA was prone to off-target events, which could be averted when two appropriately designed sgRNA sequences were used (Table S1a). A significant number of EGFP-positive cells were observed after co-transfection of WM9 and WM278 cells with sgRNA and Cas9 expression plasmids (Fig. 1c, Fig. S1). NME1-EGFP was localized primarily in the cytoplasmic compartment, identical to localization of wild-type NME1 in the respective parent cell lines, as detected by both anti-NME1 antibody and EGFP fluorescence (Fig. 1d). Thus, addition of the EGFP tag did not significantly alter the trafficking properties of NME1. Indeed, prior studies with transient expression of a similar recombinant NME1-EGFP construct confirmed a pattern of cytoplasmic localization^24,25^.

EGFP-positive cells were subjected to single cell cloning, followed by expansion and DNA sequencing to verify precise incorporation of the EGFP sequence at the endogenous *NME1* gene. Clones were identified with either heterozygous or homozygous incorporation of the EGFP DNA insert, and these genotypes were reflected in their profiles of protein expression as determined by immunoblot analysis with anti-NME1 antibody (Fig. 1e, Fig. S2). Clones expressing only the NME1-EGFP fusion protein did not harbor smaller molecular weight species, consistent with little or no proteolytic excision of the EGFP tag. To maximize EGFP fluorescence in cell subpopulations, we conducted all experiments with clones homozygous for the NME1-EGFP module. Unless otherwise specified, experiments were conducted with two clones from both the WM9 (clones 11 and 21) and WM278 (clones 2 and 6) cell lines that were homozygous for the NME1-EGFP construct.

Insertion of the EGFP sequence did not significantly impact steady-state concentrations of NME1-EGFP transcripts in the WM9 and WM278 clones, as compared with expression of native NME1 mRNA in the corresponding parental lines (Fig. 1f). As predicted, flow cytometry analysis of EGFP expression in the NME1-EGFP-expressing clones revealed heterogenous profiles of expression, with a small subpopulation of cells in each clone displaying low amounts of EGFP fluorescence (NME1^*LOW*^; Fig. 1g). To confirm the heterogeneous expression profile of NME1-EGFP, clones were sorted into three categories based upon EGFP expression (*NME1*^*LOW*^, *NME1*^*MED*^, and *NME1*^*HIGH*^; Fig. 1g). Immunoblot analysis showed that NME1^*LOW*^ cells exhibited undetectable levels of either wild-type NME1 or NME1-EGFP, indicating that the low levels of EGFP shown by flow cytometry was reflected in expression of the NME1-EGFP fusion protein (Fig. 1h, Fig. S4).

To assess the durability of NME1 expression phenotypes in subpopulations of NME1-EGFP- expressing clones, the subpopulations were isolated by FACS, expanded, and their NME1-EGFP expression monitored over several passages. NME1^*LOW*^ populations retained their low protein expression for at least 10 passages when grown under monolayer conditions (Fig. 1i). Some cells with higher levels of NME1-EGFP expression appeared within NME1^*LOW*^ cultures over the course of extended passaging, but the percentage of EGFP-positive cells remained very low (~1%). In contrast, NME1^*MID*^ and NME1^*HIGH*^ subpopulations reverted to the full NME1-expression profile characteristic of the unsorted population within 4–5 passages (Fig. S5). This indicated that melanoma cell lines are programmed to maintain a low but consistent percentage (0.5–1%) of NME1^*LOW*^ cells. Taken together, these observations indicated that expression of the NME1-EGFP fusion protein recapitulated the heterogeneous expression profile of native NME1, providing a robust approach for acquisition of viable melanoma subpopulations and assessments of their malignant potential.

### NME1^*LOW*^ cells exhibit unaltered rates of cell proliferation in monolayer culture but are self-adhesive and highly invasive in three-dimensional (3-D) systems

**Figure 2:**
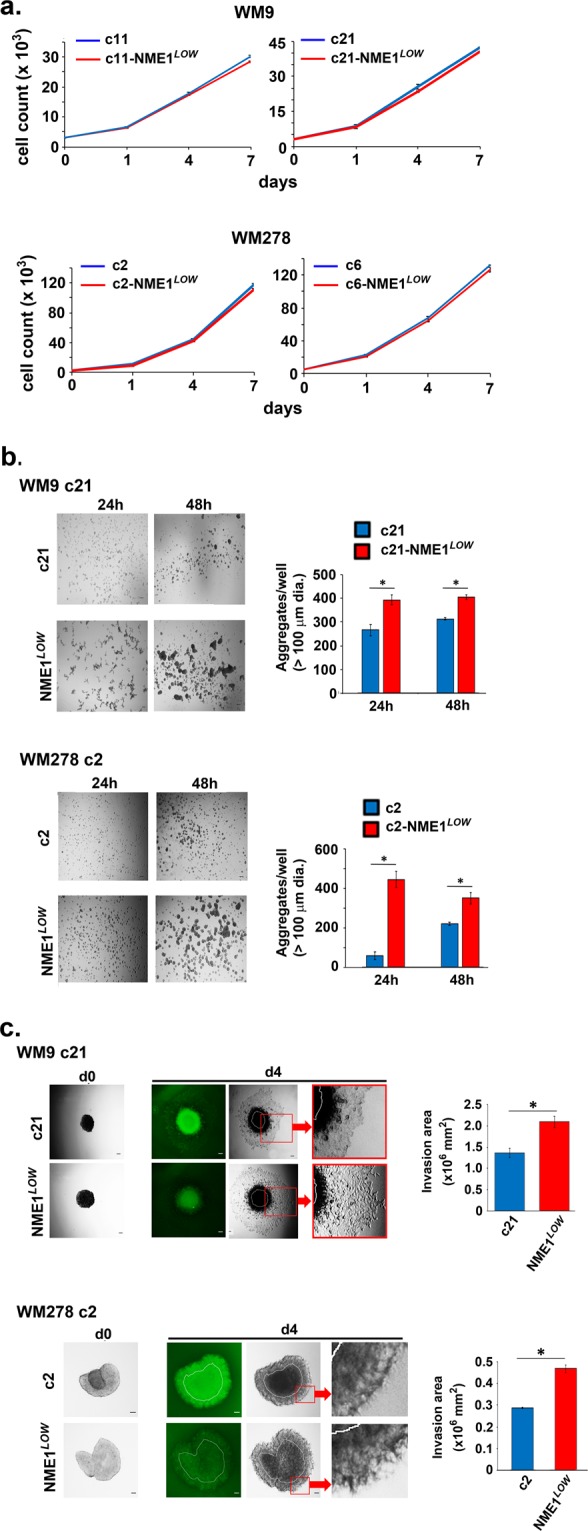
Melanoma cell subpopulations expressing low amounts of NME1-EGFP (NME1^*LOW*^) exhibit unaltered rates of proliferation under two-dimensional culture conditions, but are self-adhesive and exhibit highly invasive character in three-dimensional culture. (**a**) Shown are cell counts obtained over the indicated time course (0–7d) for parental (red lines) and NME1^*LOW*^ (blue lines) subpopulations from WM9 (clones 11 and 21) and WM278 (clones 2 and 6) cell lines (n = 5/time point/clone). (**b**) Displayed at left are representative images of cell aggregates obtained over 48 h in non-adherent culture conditions with parental (red lines) and NME1^*LOW*^ (blue lines) subpopulations from WM9 (clone 21) and WM278 (clone 2) cell lines (scale bar: 100 μm) Aggregate numbers (>100 μm diameter) obtained with the subpopulations are quantified at right. (**c**) Shown are images (left panels) and quantification of invasion areas (right panels) of cells through Matrigel in a 3D invasion assay. Cell aggregates were formed under non-adherent conditions, implanted in Matrigel, and monitored for the indicated time periods, as described in Methods. Results are shown from parental and NME1^*LOW*^ subpopulations of the WM9 (clone 21) and WM278 (clone 2) lines.

NME1^*LOW*^ cells derived from NME1-EGFP-expressing clones derived from the WM9 and WM278 cell lines displayed identical rates of proliferation as their unsorted parental counterparts when cultured as monolayers on an adherent plastic surface (Fig. 2a). This observation is consistent with many prior studies demonstrating that modulating expression of NME1 does not impact proliferation of cells in 2-dimensional culture systems^14,26^. However, when seeded into plastic wells with non-adherent surfaces, NME1^*LOW*^ cells formed 3-D aggregates more rapidly than cells from the respective parental clones (Fig. 2b). Self-aggregation has been associated with increased tumorigenicity^27^, collective migration^28^, and resistance to anoikis^29^. In addition, NME1^*LOW*^ cells isolated from both WM9- and WM278-derived clones displayed greatly enhanced invasive activity when transplanted as pre-formed spheroids into Matrigel, with invasion occurring predominantly in a collective mode (Fig. 2c).

### Xenografts generated from NME1^*LOW*^ cells in immunodeficient (NSG) mice display unaltered rates of tumor growth but greatly enhanced metastatic activity to lung and liver tissues

**Figure 3:**
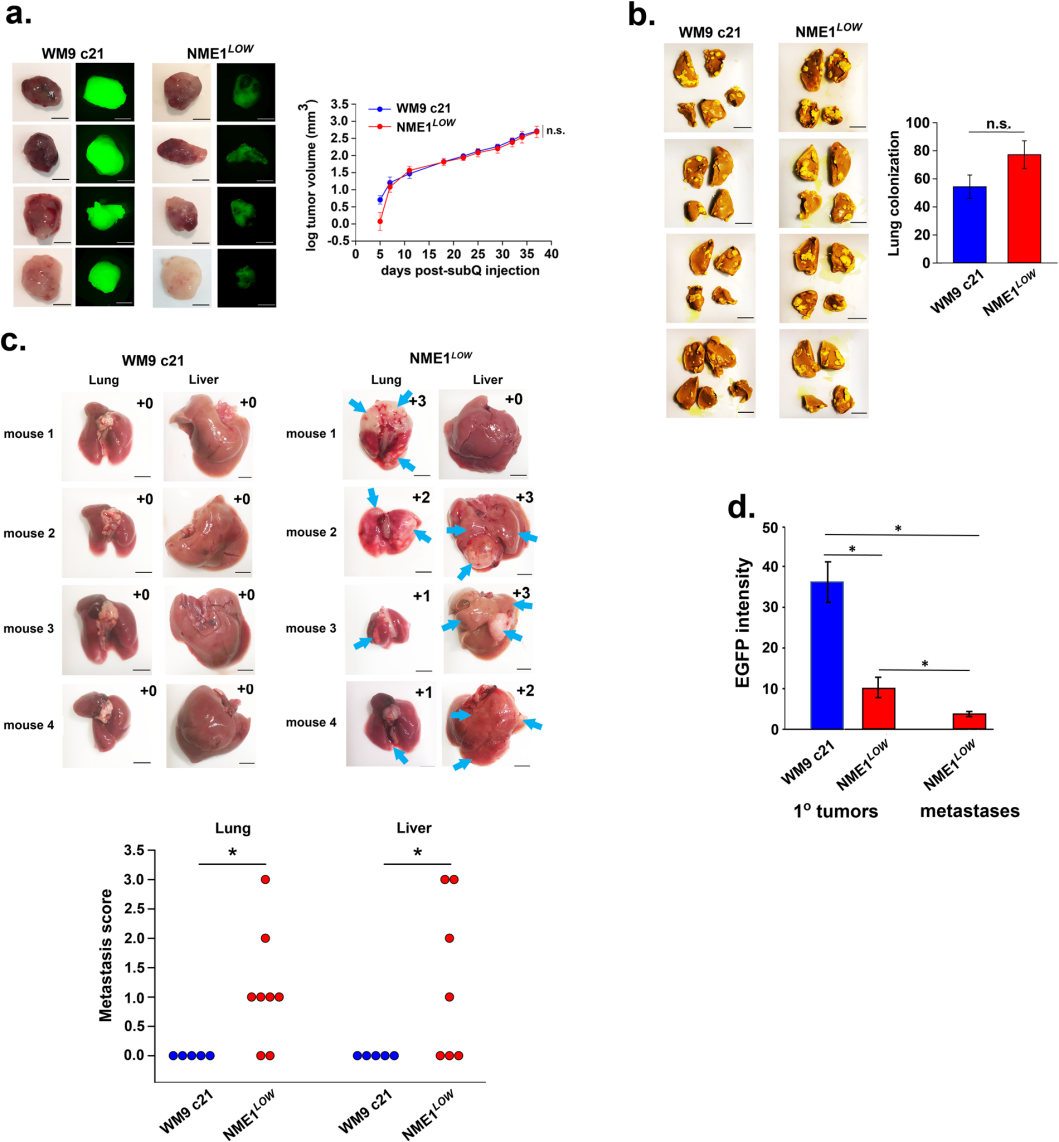
NME1^*LOW*^ subpopulations derived from human melanoma cell lines exhibit unaltered growth as primary tumor xenografts but are highly metastatic from a primary tumor location (spontaneous metastasis). (**a**) Shown at left are representative color and EGFP fluorescence images of subcutaneous tumor xenografts obtained in NSG mice from parental WM9 clone 21 (n = 12) and the corresponding NME1^*LOW*^ subpopulation (n = 13). Scale bar: 5 mm. Tumor growth rates of the two cell lines are summarized at right (mean ± SEM). n.s., non-significant by t-test. (**b**) The NME1^*LOW*^ subpopulation is unaltered in its ability to colonize lung tissue following injection via the tail vein (experimental metastasis). Injections were performed in NSG mice with cells (10^5^) from the parental WM9 clone 21 (total n = 8) or the corresponding NME1^*LOW*^ subpopulation (total n = 9). Representative images of lungs stained with Bouin’s fixative are shown at left. Scale bar: 5 mm. Lung colonization activity (right panel) was quantified as a composite score based on total number and size of visible metastatic lesions (Methods). n.s., non-significant by two-tailed t-test. (**c**) NME1^*LOW*^ cells metastasize aggressively to lung and liver from subcutaneous melanoma xenografts. Xenografted tumors in mice of panel (a) were surgically removed upon reaching a volume of 500 mm^3^, with mice sacrificed at 8 weeks post-surgery. Representative images of lungs and livers are shown in the upper panels, with composite metastasis scores (scale 0–4) are included within each panel. Dot plots displayed in the lower panel summarize metastasis scores obtained for all mice. Asterisks denote significant differences between groups, as analyzed by Mann-Whitney rank sum test (p < 0.05). (**d**) Quantification of EGFP intensity for primary and metastatic tumors. Asterisks denote significant differences between groups (t- test; p < 0.05).

To assess the tumor growth activity of NME1^*LOW*^ cells *in vivo*, unsorted and NME1^*LOW*^ cells from WM9 clone 21 (WM9c21) were injected subcutaneously into NSG mice. Both cell preparations elicited melanoma lesions in all mice within seven days of injection, with nearly identical rates of rapid tumor growth observed (Fig. 3a). Tumors obtained with NME1^*LOW*^ cells displayed much lower EGFP fluorescence than tumors from the parent clone, indicating retention of their NME1^*LOW*^ phenotype over the course of the study. No significant difference (P = 0.10) was detected between the cell preparations in generation of colonies within the lung following injection via the tail vein (experimental metastasis; Fig. 3b). To compare the ability of the two cell preparations to carry out the full metastatic cascade, mice in panel a that were injected subcutaneously with parental WM9c21 or NME1^*LOW*^ cells were monitored for an additional eight weeks after surgical excision of their primary xenografts (spontaneous metastasis assay). Primary xenografts obtained with parental cells gave rise to no visible metastatic lesions after the eight-week incubation period in any of the tissues examined (i.e. lung, liver, internal viscera, skin). In contrast, the majority of xenografts from NME1^*LOW*^ cells gave rise to metastases in both lung (6/8 mice) and liver (4/7 mice)(Fig. 3c). Those metastatic lesions retained their low level of EGFP expression, demonstrating that the NME1^*LOW*^ phenotype was stable throughout the 15-week course of the *in vivo* metastasis study (Fig. 3d).

### NME1^LOW^ cells display a unique profile of mRNA expression consistent with a neural crest-like phenotype

Previous studies have analyzed heterogenous expression patterns found within melanoma tumors and identified specific genes associated with melanoma virulence. For example, a gene expression program associated with MITF^LOW^/AXL^HIGH^ expression has been associated with decreased survival and increased resistance to therapy^19^. In addition, JARID1B-expressing subpopulations within melanoma cell lines were vital for maintained tumor growth, with slow-cycling JARID1B-positive cells displaying enhanced spontaneous metastasis activity^18,20^. However, expression of MITF, AXL and JARID1B proteins was not different between parental and NME1^*LOW*^ cell preparations from the WM9c21 and WM278c2 clones (Fig. S6), demonstrating that the NME1^*LOW*^ subpopulation is distinct from the previously described MITF^LOW^/AXL^HIGH^ and JARID1B-high subpopulations. Expression of canonical melanoma stem cell marker proteins (SOX2, OCT4, KI67) was also similar between the cell preparations (Fig. S7), strongly suggesting the NME1^*LOW*^ subpopulation does not possess increased stemness relative to the parent population. Together, these observations strongly suggested that the NME1^*LOW*^ subpopulation represents a unique and virulent entity within melanoma cell cultures.

**Figure 4:**
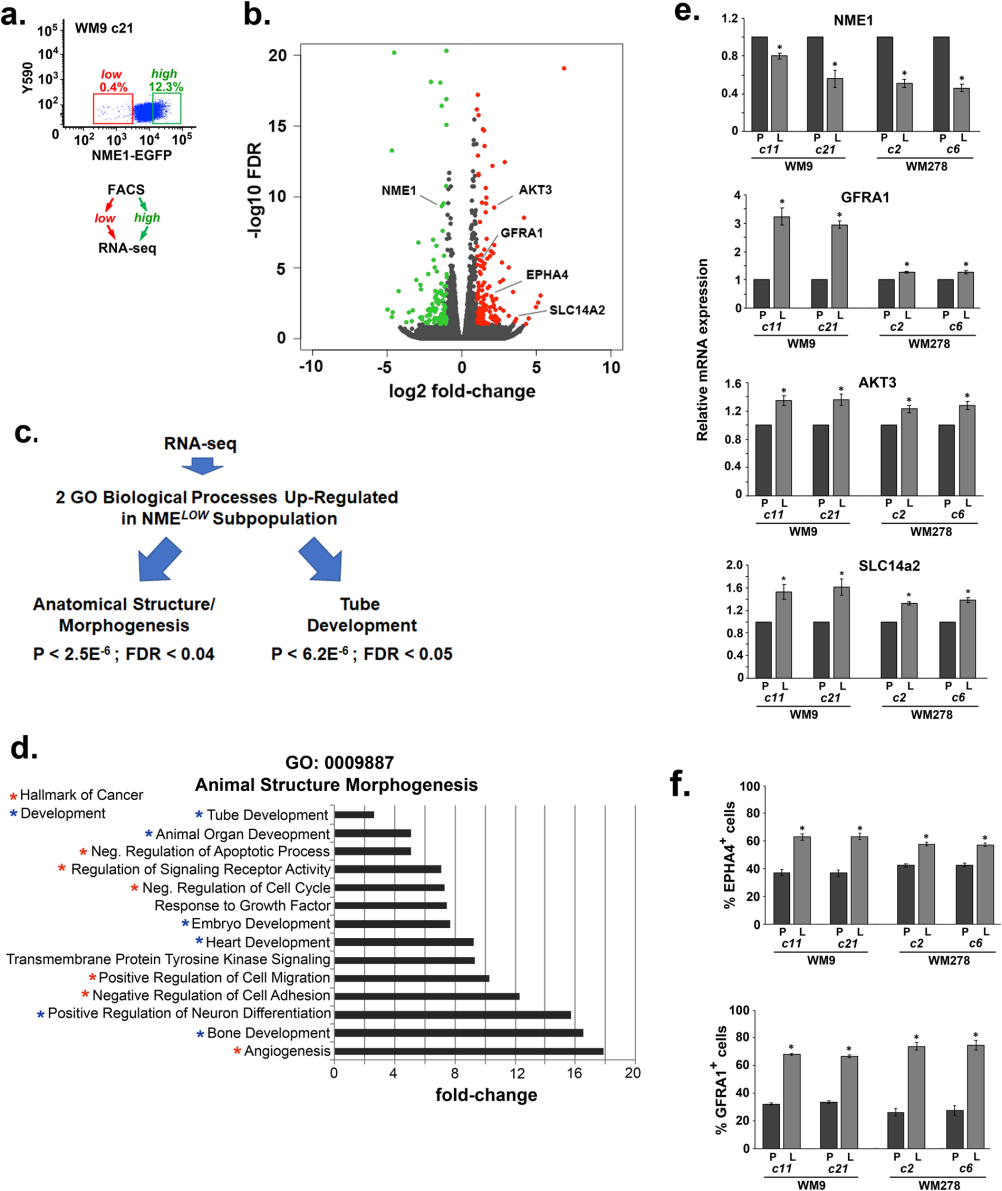
The NME1^*LOW*^ subpopulation exhibits a gene expression profile consistent with a neural crest-like phenotype. (**a**) Isolation of NME1^*LOW*^ and NME1^*HIGH*^ subpopulations from WM9 clone 21 by FACS for RNA-seq analysis. (**b**) Volcano plot of mRNA expression in NME1^*LOW*^ vs. NME1^*HIGH*^ subpopulations. (**c**) Flow chart of steps involved in identification of two GO biological processes that were upregulated in the NME1^*LOW*^ subpopulation. (**d**) Processes associated with the biological process anatomical structure/morphogenesis. Processes identified as *“Hallmarks of Cancer”* or associated with *“Development”* are highlighted with red or blue asterisks, respectively. (e) Validation of RNA-seq analysis across multiple NME1-EGFP-expressing clones. Steady-state expression of mRNAs encoding NME1, GFRA1, AKT3 and SLC14a2 was determined by qRT-PCR in the indicated parental clones (“P”) and NME1^*LOW*^ (“L”) subpopulations. Asterisks denote significant differences between matched parental clone and NME1^*LOW*^ (“L”) subpopulations, as determined by paired t-test (p < 0.05) (f) Inductions in expression of RNAs encoding EPHAA4 and GFRA1 are reflected in expression of the cognate cell surface proteins at the cell surface (p < 0.05, t-test).

To identify phenotypic markers associated with the NME1^*LOW*^ subpopulation, NME1^*LOW*^ and NME1^*HIGH*^ subpopulations were obtained by FACS from the WM9c21 cell line and subjected to RNA-sequencing (RNA-seq; Fig. 4a). 229 genes were found to be differentially expressed between the two cell preparations, of which 127 genes were upregulated in NME1^*LOW*^ cells and 102 downregulated (Fig. 4b, Table S2a). Consistent with the direct measurements of stemness-related proteins (Fig. S71), no differences in expression were observed across a wide spectrum of mRNAs encoding known melanoma stem cell genes (e.g. CD34, CD44, ALDH1, JARID1, NANOG, SOX2, SOX10, OCT4, KLF4, CCNB1 *et al*.)(Table S3). As expected, NME1 mRNA was significantly downregulated in NME1^*LOW*^ cells, as was its homolog NME2 (Table S2a). The mRNA expression profile of NME1^*LOW*^ cells was correlated with two Gene Ontology (GO) “biological processes”, anatomical structure/morphogenesis and tube development (Fig. 4c, Table S2b). No GO categories were correlated with low gene expression in NME1^*LOW*^ cells. Within the ontological process of anatomical structure/morphogenesis, fourteen related processes were identified (Fig. 4c, Table S2c) and most of these had clear relevance to metastatic potential (e.g. positive regulation of cell migration, negative regulation of cell adhesion, angiogenesis). Thirty-seven individual genes relating to anatomical structure/morphogenesis (Table S2d) and 15 tube development-related genes (Table S2e) were significantly upregulated in NME1^*LOW*^ cells. The mRNA expression profile of NME1^*LOW*^ cells was also associated with processes relating to embryonic development of neurons, heart and bone (Fig. 4d, Table S2c). The neural crest origin of these three distinct tissues strongly suggests the NME1^*LOW*^ subpopulation possesses a more neural crest-like phenotype than the NME1^*HIGH*^ subpopulation.

To validate the RNA-seq approach and determine the extent to which these expression profiles were also seen in other melanoma clones, mRNA expression patterns of NME1 and three other differentially-expressed genes was measured by qRT-PCR across other WM9- and WM278-derived cell lines. Genes were chosen for validation by qRT-PCR corroboration based upon strong (SLC14a2), relevance to significant pathways (AKT3), or its identification as a regulated cellular component (receptor: GFRA1). The relative gene expression trends obtained by qRT-PCR (Fig. 4e) reflected those obtained in the RNA-sequencing analysis. While expression of the NME1 transcript was indeed lower in NME1^*LOW*^ subpopulations derived from four different clones, the decreases in each clone were rather modest. This is consistent with our prior demonstration that NME1 protein expression is suppressed in melanoma cells primarily due to lysosome-mediated degradation of the protein and not suppression of the NME1 transcript^30^.

NME1^*LOW*^ cells also displayed upregulation of the GO category, “cellular components”, of which the classification “receptor complexes” was identified (Table S2f-g). Expression of two receptor proteins (EPHA4, GFRA1) associated with the neural crest phenotype^31^ and whose transcripts were upregulated in the NME1^*LOW*^ subpopulation of WM9c21 cells were evaluated by immunostaining and flow cytometry. While both unsorted and NME1^*LOW*^ subpopulations expressed, the proportion of EPHA4- and GFRA1-positive cells was significantly higher in NME1^*LOW*^ cells (Fig. 4f). This verified that the elevated expression for EPHA4 and GFRA1 mRNAs in NME1^*LOW*^ cells is indeed manifested by an increase in cells with elevated expression of the cognate proteins at the cell surface. Importantly, it also indicates the NME1^*LOW*^ subpopulation is enriched for cells with a neural crest-like phenotype.

## Discussion

Cell subpopulations present with a primary tumor are not equally susceptible to current therapeutic measures^2–4^. As metastasis is the primary cause of cancer-related death^1,8,32^, it remains crucial to identify highly metastatic subpopulations that can be targeted to prevent recurrence and progression of malignant disease. Through analysis of the protein expression pattern for the metastasis suppressor protein, NME1, we have discovered a previously unidentified subpopulation present within melanoma cell lines that is highly metastatic and may well represent a barrier to successful therapy in melanoma patients.

As a metastasis suppressor, NME1 is defined by the ability to inhibit metastasis, while having no impact on initial formation or growth of tumors^8,33,34^. Consistent with that definition, proliferation rates of NME1^*LOW*^ cells *in vitro* were not different from their unsorted parent cell lines but they did exhibit greater invasive activity. Moreover, NME1^*LOW*^ cells did not exhibit an increased ability to induce lung colonization using the tail vein injection approach, which bypasses initial steps of the metastatic cascade. Using the spontaneous metastasis approach which recapitulates the full metastatic process, however, NME1^*LOW*^ cells were highly metastatic to both lung and liver tissues. This observation stood in marked contrast to the unsorted parental cells, which did not give rise to any visible metastases. Taken together, these studies strongly suggest that low NME1 expression in this melanoma subpopulation confers an enhanced ability to overcome one or more steps of the metastatic cascade (e.g. tissue invasion, intravasation, emboli formation) that precede the barriers of extravasation and colonization. Importantly, they identify NME1^*LOW*^ subpopulation as a rare but highly metastatic entity that is likely to exist in human melanoma patients and which may play a key role in tumor progression, recurrence and metastasis.

Tumor heterogeneity occurs through heritable and non-heritable variation^3,5,6^. To a large degree, the analysis of non-heritable heterogeneity has not focused on metastatic activity but rather on the identification of slow-growing cancer stem-like populations and their abilities to initiate tumors in immune-compromised mice^35^. Indeed, a prior study showed that almost all melanoma cells have the ability to initiate tumors *in vivo*^36^. However, NME1^*LOW*^ cells exhibited unaltered rates of proliferation and expression of cancer stem cell markers, as well as tumor initiation capacity *in vivo*. NME1^*LOW*^ cells are capable of self-renewal and maintain a “low” NME1 state in the face of extensive passaging in culture. In a prior study, we observed that expression of NME1 was required for maintenance of a stem-like phenotype in melanoma cells cultured under non-adherent conditions that promote spheroid growth^17^. In contrast, the current study shows that the naturally occurring NME1^*LOW*^ subpopulation seen in monolayer cultures does not exhibit altered expression of known stem cell markers. This suggests that stemness tone in the rare NME1^*LOW*^ subpopulation is maintained in the absence of NME1 expression and does not exclude the possibility that NME1 is required for maintenance of stemness in the bulk cell population.

Previous analysis of melanoma heterogeneity has led to the identification of markers with varying levels of tumor initiation and invasion capacity. We sought to determine if the NME1 subpopulations have a similar expression pattern to previously identified melanoma subgroups by analyzing protein expression of MITF, AXL, and JARID1B^18–20^, but observed no relationship between expression of NME1 and these established markers. These results, combined with no change in stem cell marker expression, suggested that our NME1^*LOW*^ cells represent a previously unidentified melanoma subpopulation. NME1^*LOW*^ cells did not exhibit altered expression of mRNAs associated with differentiated melanocytes or transcripts encoding the putative melanoma virulence factors MITF, AXL, and JARID1b. However, over 200 differentially expressed genes were identified, many of which are associated with reduced apoptosis, enhanced migration, angiogenesis, and tissue development. Of particular interest in NME1^*LOW*^ cells was the identification of an upregulated program of genes related to embryonic development of heart, neuron, and bone tissues. In light of the critical role of the neural crest in formation of these tissues, the RNA expression profile of NME1^*LOW*^ cells appears to reflect a de-differentiated phenotype. Gene ontology analysis of our RNA-sequencing results also revealed that NME1^*LOW*^ cells overexpress six RNAs associated with receptor complexes, four of which (EPHA4, GFRA1, NTRK2 and PLXNA2) play key roles in neural crest cell development. EPHA4, GFRA1 and PLXNA2 are associated with promoting neural crest cell migration, while NTRK2 allows for neural crest cell survival. GFRA1 has also been linked to cancer metastasis^37^. Interestingly, EPHA4 and NTRK2 are both receptor tyrosine kinases (RTK), while GFRA1 mediates ligand-mediated activation of another RTK, RET^31,38,39^. RTKs have proven useful targets for small molecule inhibitors and other approaches in multiple settings of cancer^40,41^, suggesting the NME1^*LOW*^ subpopulation might be susceptible to similar RTK-directed approaches. Moreover, the cell surface localization of these cell surface receptors renders these genes potential targets for novel antibody-drug conjugates (ADC). Recently, a study demonstrated that ADC-GFRA1 resulted in cytotoxicity of GFRA1 over-expressing cell lines and patient derived xenografts in breast cancer^37^. Future studies are warranted to assess the incidence of NME1^*LOW*^ subpopulations in human melanoma specimens, to determine their association with melanoma staging and patient survival, and to assess their merits as therapeutic targets.

## Methods

### Approval for experimental protocols

All experimental protocols in this study were approved by the Institutional Biosafety Committee at the University of Maryland-Baltimore (protocol number IBC-00001928). All methods were performed in accordance with relevant guidelines and regulations.

### Cell culture

Metastatic melanoma cell lines, WM9 and WM278, were gifts of Dr. Meenhard Herlyn (Wistar Institute, Philadelphia, PA, USA). Monolayer WM9 and WM278 melanoma cell lines were cultured in 5% CO_2_ at 37 °C with TU2% media. TU2% media consists of a 4:1 (v/v) ratio of MCDB:Leibovitz L-15 media (Sigma-Aldrich) and 0.1% (v/v) sodium bicarbonate (Gibco), supplemented with 2 mM CaCl_2_, 2.5 μg/ml insulin, and 2% FBS (Gibco) and adjusted to pH 7.2. Cells were cultured at 37 °C and 5% CO_2_. Cell lines were authenticated by the University of Maryland School of Medicine Center for Innovative Biomedical Resources, Genomics Core – Baltimore, Maryland.

### CRISPR design

The CRISPR design tool, CHOPCHOP (http://chopchop.cbu.uib.no/index.php)^20^, was utilized to identify the location of potential sgRNA target sites along the NME1 locus, as well as determine off-targets in the genome. A list of the potential sgRNAs for NME1 and the off-target prediction can be found in Table S1a. In order to prevent the sgRNA from recognizing and binding to the donor sequence, we designed the 5’ homologous arm of the donor sequence to contain 5 silent mutations. We utilized the Codon Adaptation Index (CAI) Calculator (Biologics Corp International, https://www.biologicscorp.com/) to analyze for codon usage bias.

### Generation of NME1-EGFP melanoma clones

Plasmids were designed (Celltechgen) to contain either Cas9^D10A^/sgRNAs (pST1374-N-Cas9-D10A-NLS-U6-sgRNA) or NME1-EGFP (pSIMPLE19-NME1-EGFP) donor sequence (Figure S1a). Plasmids were transformed into competent bacterial cells and were purified from the bacterial cultures with ZymoPURE Plasmid Maxiprep (D4203). Genomic sequencing confirmed accurate sgRNA and donor sequence prior to transfection. The donor vector was linearized by BamH1 restriction enzyme digestion at the distal end of the 5’ homologous arm, followed by purification (QIAquick PCR Purification Kit 28106) and quantification with a Nanodrop spectrophotometer (ThermoFisher. Linearized donor sequence (2 μg) and the Cas9^D10A^/sgRNA vector (2 μg) were co-transfected into two metastatic melanoma cell lines (WM9 and WM278) with the Lonza Nucleofector KitR (program P-031). Transfected cells were selected by a 72 h incubation in TU2% media containing blasticidin at a concentration of 4 μg/ml. After one week of growth, viable cells were sorted for EGFP expression (BD Aria II), followed by single cell cloning. Accuracy of NME1-EGFP incorporation was determined through genomic sequencing of the resultant clones. Primers used for genomic sequencing of NME1-EGFP are listed in Table S1b.

### Fluorescence-activated cell sorting (FACS)

For fluorescence-activated cell sorting, cells were washed 2x with PBS and incubated in 0.05% Trypsin-EDTA (Gibco) for 3 minutes at 5% CO_2_. Once cells were non-adherent, the trypsin reaction was quenched with TU2%. Cells were centrifuged at 1000 g for 3 min, washed once with PBS, and resuspended with sample buffer (2% FBS in PBS) at a concentration maximum of 10^7^cells/ml. Samples were sorted on BD Aria II into 5 mL polystyrene round-bottom FACS tubes (Corning 352058) containing collection buffer (10% FBS in PBS) and stored on ice for the duration of the sorting procedure. Viable cells for each population were counted after trypan blue staining.

### Flow cytometry

To analyze NME1 expression in NME1-EGFP expressing clones, cells were harvested as previously described in the fluorescence-activated cell sorting section of these methods. Cells were resuspended in sample buffer (2% FBS in PBS) and expression of NME1-EGFP protein was analyzed on the BD LSRII flow cytometer. For immunostaining of all untagged intracellular proteins, cells were fixed with 2% formaldehyde and the membrane was permeabilized with 0.1% (v/v) Triton X-100 in PBS. Cells were incubated with primary antibody in staining buffer (PBS, 1% (w/v) bovine serum albumin, and 0.1% (v/v) Triton X-100) for 30 min at room temperature. Cells that were exposed to an unconjugated primary antibody were washed and subsequently incubated with secondary antibody in staining buffer for another 30 min at room temperature. Cells were subjected to two washes and resuspended in sample buffer for flow cytometric analysis. Membrane-bound proteins were fixed and stained without membrane permeabilization. Cells were gated followed by quantification of mean fluorescence intensity (MFI), using FCS6 Express software for analysis of protein expression. The primary antibodies utilized for analysis are as follows: mouse monoclonal anti-NME1 (BD 610247), anti- GFRA1 antibody (ab8026), anti-EphA4 (ab5389), V450-conjugated mouse anti-Sox2 (BD 561610), AF-647-conjugated mouse anti-Oct4 (BD 560329), AF-674-conjugated mouse anti-Ki-67 (BD 561126), PE-conjugated rabbit anti-Axl (CST 78909 S), goat anti-Sox10 (sc- 17342), mouse anti-JARID1B (Ab56759), mouse anti-MITF (sc-56726). Secondary antibodies employed were: FITC Rat anti-mouse IgG (Biolegend 406001), Apc-Cy7 donkey anti-goat IgG (scH2615) and PE goat anti-mouse Ig (BD 550589).

### Immunofluorescence

Immunofluorescence was utilized to determine the localization of NME1 in both parental and NME1-EGFP cell lines. Cells were plated at 10^4^ cells in 8-well chamber slides (Lab-Tek) and were placed in a 5% CO_2_ incubator at 37 °C overnight. Adherent cells were fixed with cold methanol for 15 min at −20 °C, followed by permeabilization with 0.2% Triton X-100 and 1% normal goat serum (NGS) in PBS for 5 min at room temperature. Primary antibody suspended in dilution buffer (1% NGS in PBS) incubated with fixed cells overnight in a humidity chamber at 4 °C. Primary antibody was removed and cells were washed twice with 1% NGS in PBS. Cells were exposed to secondary antibody in dilution buffer for 1 h at room temperature. After two more washes, the chamber was removed and cover slips mounted with SlowFade Gold + DAPI (Life Tech). Slides were immediately imaged on a Leica DMi8 microscope or stored at −80 °C. The primary antibody used for imaging was NME1 (BD 610247) and the secondary antibody was donkey anti-mouse AF568 (Invitrogen A10037).

### Immunoblotting

Cells were collected and spun down into a pellet at 1000 g for 3 min. Whole cell lysates were generated by resuspending the pellets in RIPA buffer (10 mM Tris-HCl pH 7.5, 1 mM EDTA, 0.5 mM EGTA, 1% Triton X-100, 0.1% sodium deoxycholate, 0.1% SDS, 140 mM NaCl), which was supplemented with 1x Halt Protease Inhibitor Cocktail (Thermo Scientific). Protein lysates were quantified by BCA assay (Thermo Scientific) and resolved by SDS-polyacrylamide gel electrophoresis (AnyKD Criterion Precast Protein Gel, Bio-Rad), followed with transfer to nitrocellulose membrane (Bio-Rad). Membranes were blocked for 1 h at room temperature with 5% non-fat dry milk in Tris buffered saline with 0.1% Tween-20 (TBST). Subsequently, membranes were incubated overnight at 4 °C with mouse monoclonal anti-NME1 (BD 610247), diluted 1:3000 in TBST. Similarly, membranes were incubated in mouse monoclonal anti-TATA box Protein (anti-TBP, Millipore, SL30-3-563) at a 1:500 dilution to authenticate equal loading of the protein lysate. Three 10 min washes were conducted with TBST, followed by a 1 h incubation with a HRP-conjugated secondary antibody, ECL-conjugated anti-mouse IgG (1:10,000; GE Healthcare NA931V). Membranes were incubated in Amersham ECL Prime Western Blot Detection Reagent for 1 min at room temperature prior to detection on Amersham Hyperfilm ECL (GE Healthcare).

### Proliferation assay

Cell populations were plated at 3000 cells per well in a 6-well dish. Cells were collected on days 1, 4, and 7 through trypsinization and counted on a hemocytometer with trypan blue staining.

### Cell aggregation assay

Cell culture plates were coated with poly-HEMA (Sigma p3932) and allowed to dry overnight before plating of cells. Adherent WM9 and WM278 cells were harvested with 0.05% trypsin and were plated in melanoma sphere medium (DMEM/F12 (1:1; Gibco), 1% (w/v) methylcellulose (Sigma), 0.4% (v/v) bovine serum albumin (Gibco), 0.12% (v/v) sodium bicarbonate and supplemented with B27 serum-free supplement (1× ; Invitrogen), 20 ng/ml EGF, and 4 μg/ml insulin. Cells were plated at 1000 cells/well in 96-well plates (0.32 cm^2^/well; Corning).

### 3D invasion assay

96-well flat-bottom plates were coated with 50 μl of 0.75% agarose to facilitate aggregation formation. Sterile 0.75% agarose was made by autoclaving a mixture of 3.75 g of molecular grade agarose (Invitrogen) in 500 mL dH_2_O. Cells were plated at 10,000 cells per well in a total volume of 100 μl of media and incubated overnight at 37 °C and 5% CO_2_. Aggregates were embedded into a flat-bottom 96-well plate that was coated with a 1:1 (v/v) ratio of cold Matrigel and TU2%. Upon embedding, the Matrigel and TU2% mixture was allowed to solidify at 37 °C and 5% CO_2_. After 30 minutes, 100 μl of warmed TU2% was added to each well. Aggregates were monitored for invasion by microscopy at specific time intervals and imaged at 4x magnification under transmitted light (EVOS FL Imaging System, AMF4300).

### *In vivo* mouse experiments

Mouse care, injection and surgery protocols were approved by and carried out in accordance with the relevant guidelines and regulations of the Institutional Animal Care and Use Committee (IACUC) at the University of Maryland-Baltimore (protocol numbers 0515008 and 0418008). For experimental metastasis assay, cells were grown to 80% confluency and removed from the dish with enzyme-free cell dissociation buffer (Gibco). Cells were centrifuged for 3 min at 1000 rpm and washed 2 x with PBS. After counting with trypan blue, 10^6^ cells/ml were resuspended in PBS. Cells were injected intravenously into the tail vein of female NOD-Scid-Gamma (NSG, 6–8 wks-of-age) mice (10^5^ cells per mouse). Mice were sacrificed at 8 wks post-injection and inspected for metastases. Animal lungs were incubated overnight in Bouin’s fixative (Sigma-Aldrich HT10132-1L) to enhance visibility of metastatic lesions. Metastasis was quantified by analyzing both number and size of macroscopic metastatic lesions observed on lung surfaces.

For spontaneous metastasis assay, WM9 cell populations were obtained after dissociation with enzyme-free dissociation buffer and washed 2 x with HBSS. Cells were resuspended in a 1:5 (v/v) ratio of Matrigel:HBSS and injected subcutaneously in the right flank of 6–8 wk female NSG mice (2 × 10^5^ cells per mouse). Primary tumors were surgically removed, and tissue was fixed with 10% neutral- buffered formalin (Sigma HT501128-4L) overnight, with subsequent storage in 70% ethanol at 4 °C. Mice were sacrificed at 8 wks post-surgery and inspected for macroscopic metastases. Lungs and livers were obtained, imaged, and stored in 10% neutral-buffered formalin.

### RNA sequencing

Cells were sorted into low and high GFP-expressing populations. 7-AAD staining immediately prior to the sort allowed for the removal of any dead cells in each subpopulation. The populations were sorted into three separate 5 ml FACS tubes containing collection buffer, with a minimum of 2 × 10^6^ cells per replicate. Cells obtained from the sorter were centrifuged for 5 min at 2000 rpm. Collection buffer was removed and the cells were washed 2x with PBS. Total RNA was extracted with RNeasy Mini Kit (Qiagen 74106) and was subjected to DNase treatment (Qiagen 79254). All RNA samples were subjected to a quality check and were run on Illumina HiSeq. 4000. RNA-seq analysis was carried out by the Informatics Resource Center, Institute for Genome Sciences, UMDSOM. Strand-specific paired-end Illumina libraries were mapped to the Human reference, Ensembl release GRCh38.92, using HiSat2 v2.0.4, with default mismatch parameters. Read counts for each annotated gene were calculated using HTSeq. The DESeq Bioconductor package (v1.5.24) was used to estimate dispersion, normalize read counts by library size to generate the counts per million for each gene, and determine differentially expressed genes between high and low samples. Differentially expressed transcripts with a FDR ≤0.05 and log_2_ fold change ≥1.0 were used for downstream analyses. Normalized read counts were used to compute the correlation between replicates for the same condition and compute the principal component analysis for all samples. Pathway analysis of the differentially expressed genes was conducted through ConsensusPathDB^40^, as previously described^41^. Gene ontology analysis was conducted for biological processes and cellular components was conducted through The Gene Ontology Resource^42–44^.

### Quantitative reverse transcriptase polymerase chain reaction (qRT-PCR)

RNA from NME1-EGFP clones and NME1^Low^ subpopulations was extracted with the RNeasy Mini Kit (Qiagen 74106). High Capacity cDNA Reverse Transcription Kit (Applied Biosystems) was used to generate cDNA from 1 μg of purified RNA. cDNA was used directly for qRT- PCR or stored at −20 °C. cDNAs and oligodeoxynucleotide primers (Table S4) were added to 2x SYBR Green qPCR master mix (Applied Biosystems). qPCR was performed on a CFX Real-Time PCR Detection System (Biorad Laboratories), with a program set for 40 cycles of: 15 sec at 95 °C, 30 sec at 60 °C, and 5 sec at 60 °C. Expression was normalized to a control gene, RPL13a. Results were obtained from three independent experiments, which were completed in triplicate.

### Statistical analysis

Statistical significance was determined using t-test analyses executed in Microsoft Excel (Version 15.38). All data are representative as n ≥ 2 independent experiments, unless noted otherwise in figure legends. For all tests, p-values ≤ 0.05 were considered statistically significant.

## Supplementary information

Supplementary Information.

## Data Availability

Datasets generated during the current study are available in the NCBI Gene Expression Omnibus [https://www.ncbi.nlm.nih.gov/geo/query/acc.cgi?acc = GSE140150]. All other data generated or analysed during this study are included in this published article (and its Supplementary Information files). All materials, data and associated protocols will be made available promptly without preconditions.
