# Supplementary material for: A rare subpopulation of melanoma cells with low expression of metastasis suppressor NME1 is highly metastatic in vivo
Source: Sci Rep. 2020 Feb 6;10:1971. doi: 10.1038/s41598-020-58996-3 (PMC7005181; doi:10.1038/s41598-020-58996-3)
Supplement: Supplementary file 1 — Supplementary Information. [file 41598_2020_58996_MOESM1_ESM.pdf]

Supplementary Information

A rare subpopulation of melanoma cells with low expression of metastasis suppressor NME1 is highly metastatic *in vivo*

Devin Snyder, Ying Wang and David M. Kaetzel

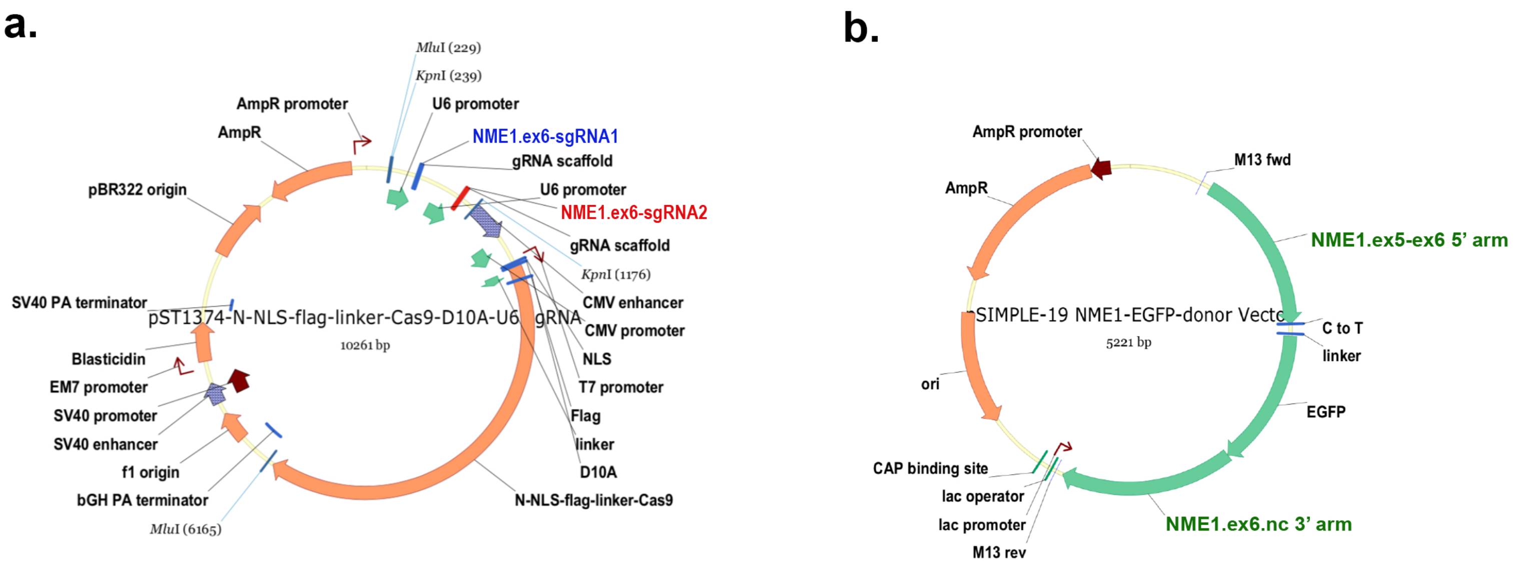

Supplementary Figure 1. (**a**) Map of pST1374-N-NLS-flag-linker-Cas9-D10A-U6 gRNA used for expression of sgRNAs 1 and 2 (highlighted in blue and red font, respectively), gRNA scaffold, and Cas9. (**b**) Map of p SIMPLE-19 NME1-EGFP donor vector. Highlighted within the map are the 5' arm derived from the junction of exons 5 and 6 of the NME1 gene and the 3' arm derived from non-coding (nc) sequence from exon 6.

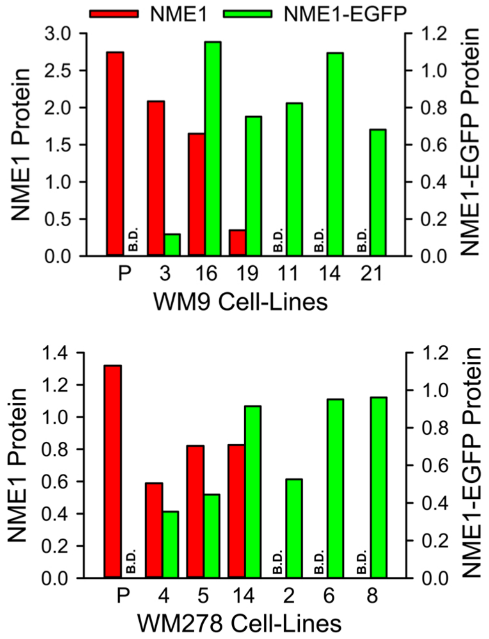

Supplementary Figure 2. Quantification of immunoblot images in Fig. 1e. B.D., below detection. Intensities of bands corresponding to native NME1 (19 kDa) and the NME-EGFP fusion protein (47 kDa) were quantified using ImageJ.

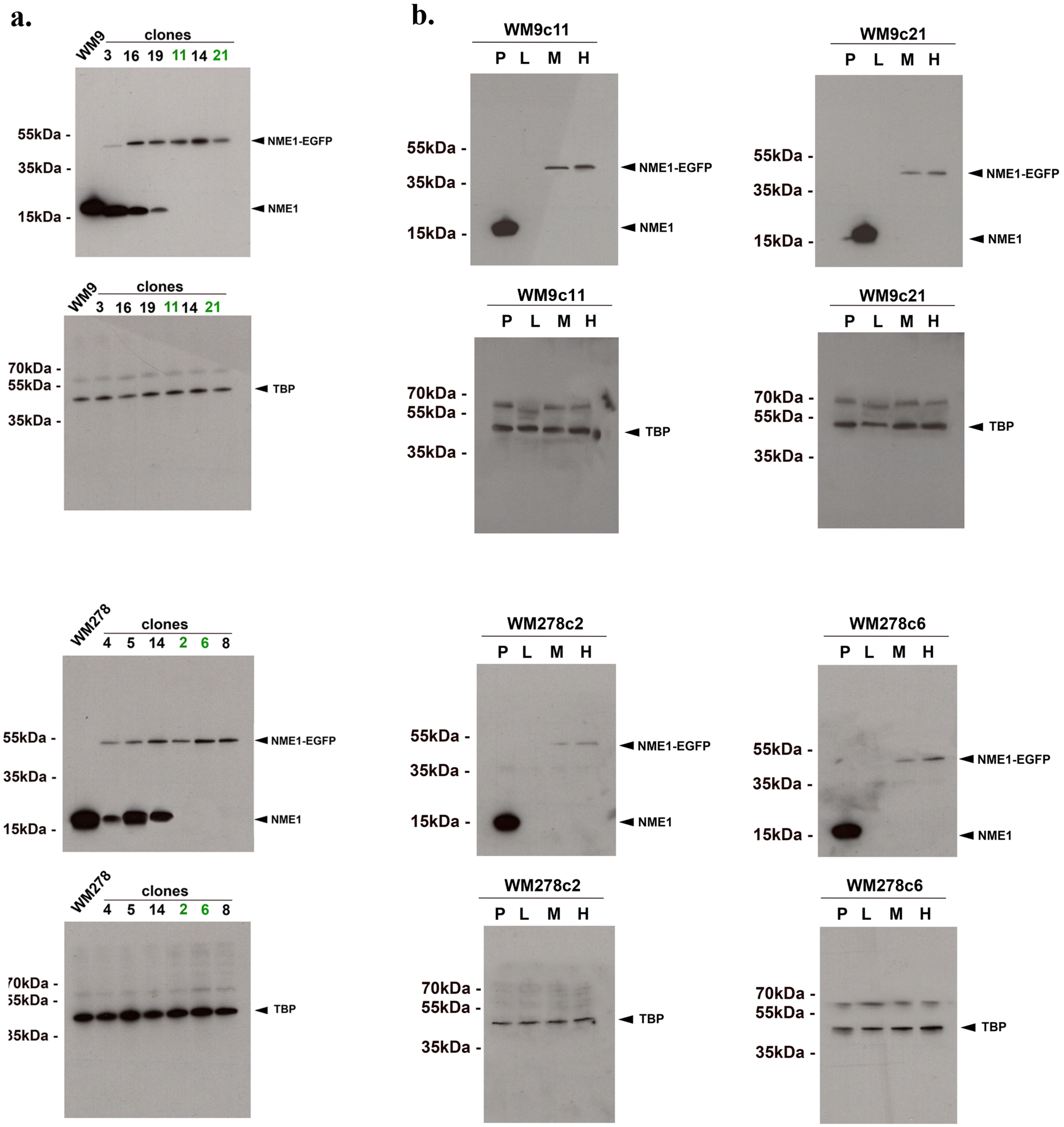

Figure S3. (**a**) Images of full-size immunoblots corresponding to data shown in Figure 1e. (**b**) Images of full-size immunoblots corresponding to data shown in Figure 1h.

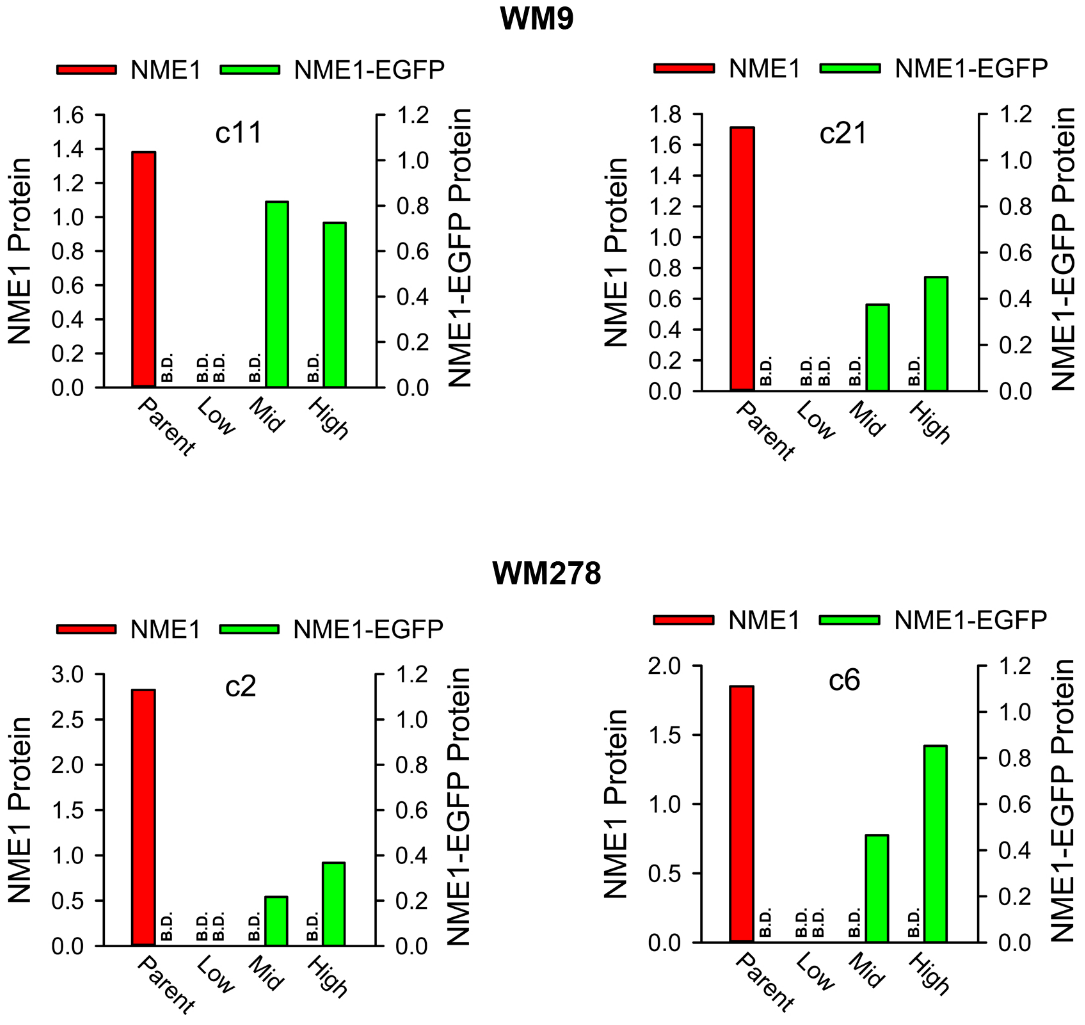

Supplementary Figure 4. Quantification of immunoblot images in Fig. 1h. B.D., below detection. Bands corresponding to native NME1 and the NME1-EGFP fusion protein were quantified using ImageJ.

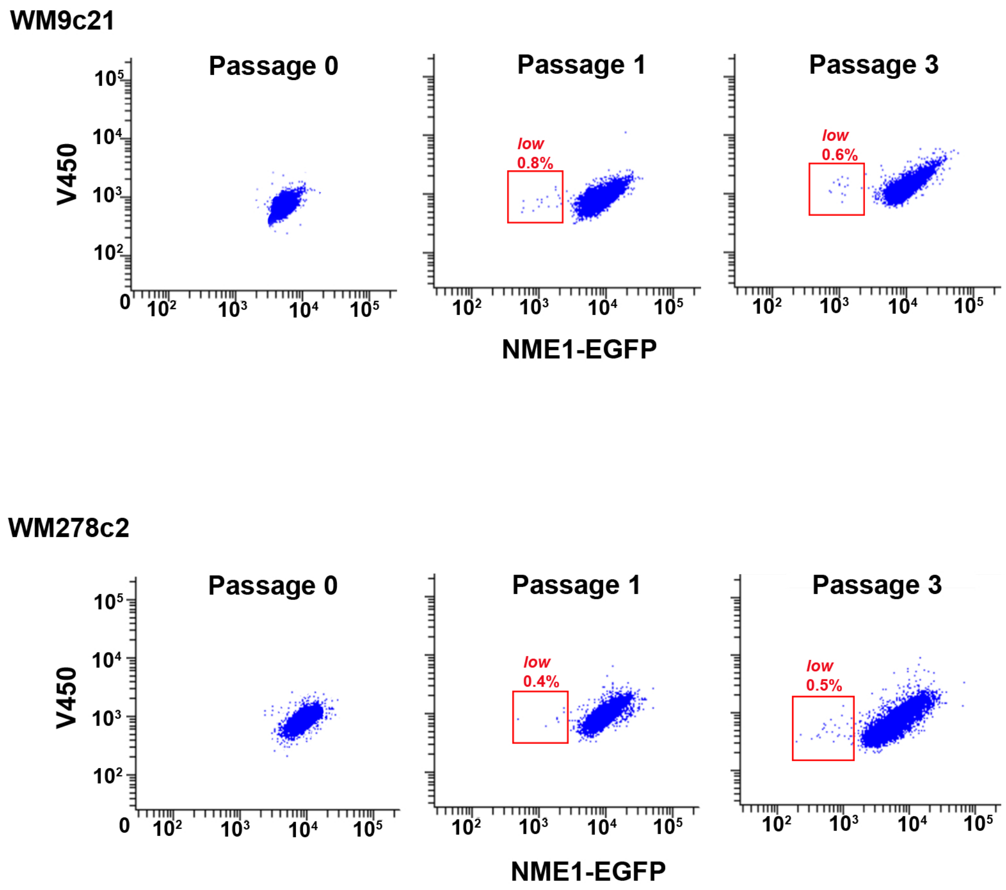

Supplementary Figure 5. NME1^{HIGH} subpopulations from WM9c21 and WM278c2 re-establish low-abundance NME1^{LOW} subpopulations after passaging in monolayer culture. Red boxes identify NME1^{LOW} cells in the respective cultures.

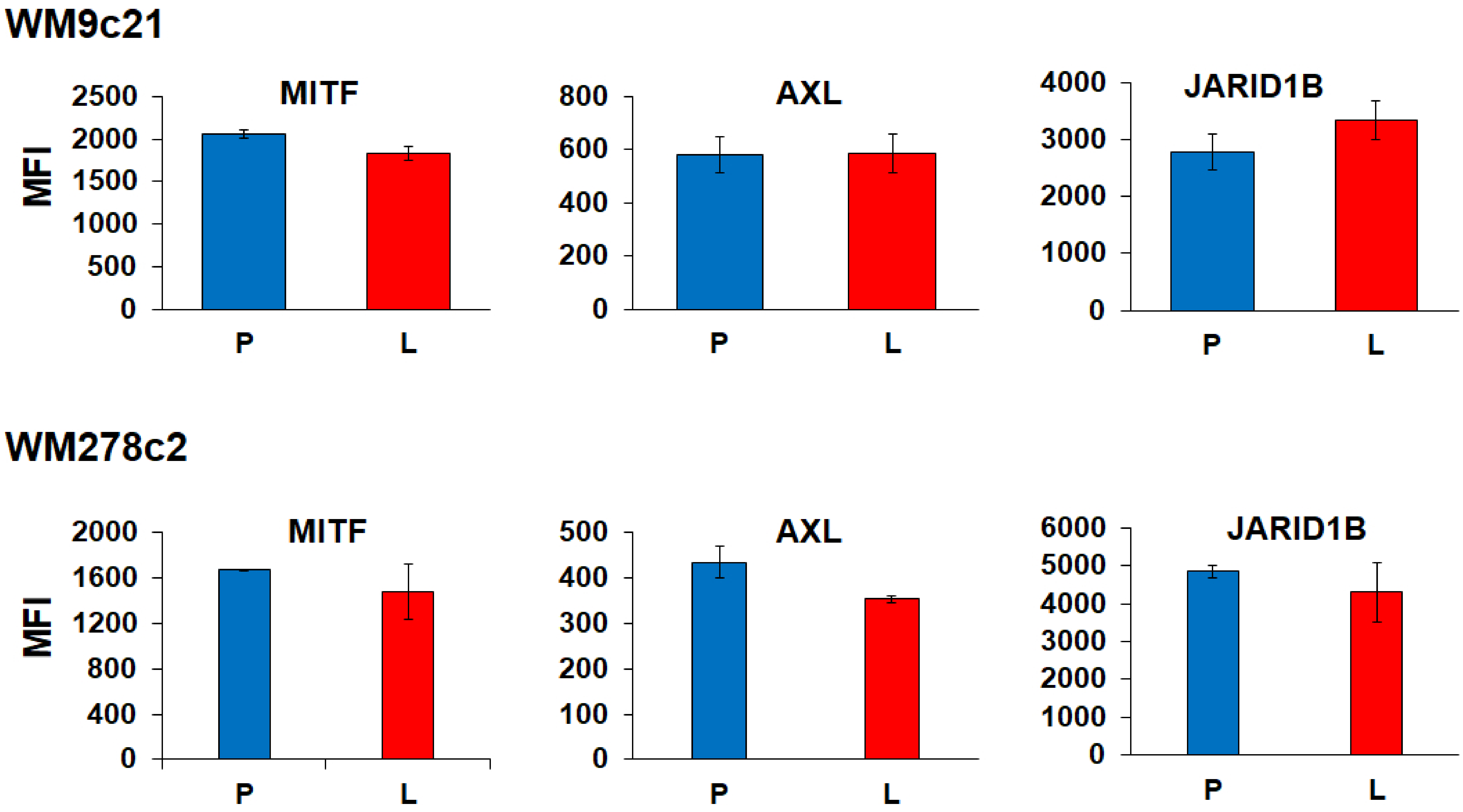

Supplementary Figure 6. Expression of MITF, AXL and JARID1B is unaltered in NME1^{LOW} subpopulations from WM9c21 and WM278c2 cells. *P*, parental/unsorted cells; *L*, NME1^{LOW} cells. Mean fluorescence intensity (MFI) represents the average expression of the interrogated proteins on a per cell basis, determined as described in Methods.

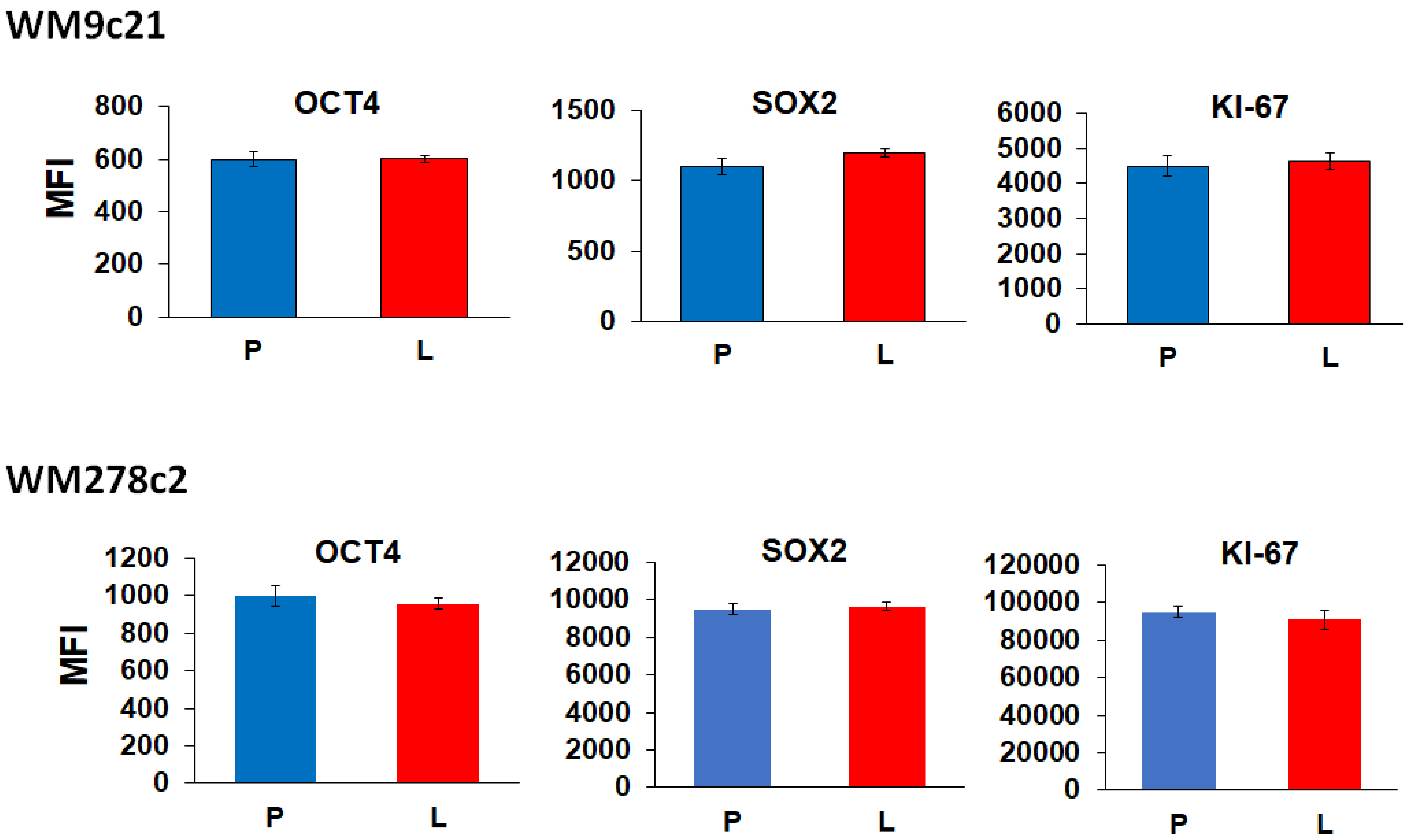

Supplementary Figure 7. Expression of stem cell markers OCT4, SOX2 and KI67 is unaltered in NME1^{LOW} subpopulations from WM9c21 and WM278c2 cells. P, unsorted parental cells; L, NME1^{LOW} cells.

Table S1A. sgRNA sequences used for CRISPR-Cas9-mediated insertion of EGFP coding sequence into the genomic *NME1* locus.

sgRNA	Sequence (5' - 3')
NME1.ex6-sgRNA1	**CCT**GAGGAACTGGTAGATTACAC
NME1.ed6-sgRNA2	GAACTGGATCTATGAATGAC**AGG**

Note: Nucleotides in blue represent the PAM sequence

Table S1B. Oligodeoxynucleotide primers used for sequencing of *NME1-EGFP* coding sequence in CRISPR-Cas9-engineered melanoma cell lines.

Primer	Sequence (5' - 3')
NME1-1F	CGGCCAGTGAATTAGAACTCGG
NME1-1R	CTTCACCACATTCAGCCCCTCC
NME1-2F	CTTCTGTCCTTGGAGGTCTGGG
NME1-2R	GCCAAACGCCAACTTAAGAGGG
NME1-3F	CCCTCTTAAGTTGGCGTTTGGC
NME1-3R	GGATTAAAATGCGCCCATTGCC
NME1-4F	GCTGTGGCTGTAGATTTCTGGC
NME1-4R	GGACACGCTGAACTTGTGGC
NME1-5F	GTAAACGGCCACAAGTTCAGCG
NME1-5R	GCCCTCGAACTTCACCTCGG
NME1-6F	GACGACGGCAACTACAAGACCC
NME1-6R	GTCTTTGCTCAGCTTGGACTGG
NME1-7F	GACAACCACTACCTGAGCACCC
NME1-7R	TTAATCAGATGGTCGGGGATGG
NME1-8F	TCTTGGAGCTGTGAGTTCTCCC
NME1-8R	TCTGCACCTAGTCCAGTCTTGG
NME1-9F	GGCACTGAGCTATTCAACAGGC
NME1-9R	ACCCTGGACTTGAACTTCTGGC
NME1-10F	AGAAGTTCAAGTCCAGGGTGGG
NME1-10R	TGATTACGCCAAGTTTGCACGC

Table S2A. List of genes differentially expressed in the NME1^{LOW} subpopulation.

Gene	Log Fold Change	P-Value	False Discovery Rate
MTATP6P1	1.01	2.88E-20	6.72E-17
SLIT2	1.01	1.17E-03	4.51E-02
GPRC5A	1.02	1.68E-05	1.74E-03
FAM3C2	1.03	7.98E-10	3.07E-07
OLFML2B	1.04	5.22E-06	6.59E-04
RPL9P7	1.04	4.80E-09	1.54E-06
EDIL3	1.05	2.18E-08	6.21E-06
PLXNA2	1.07	5.49E-06	6.89E-04
MRGPRX4	1.07	1.04E-05	1.17E-03
FBL	1.07	2.28E-04	1.36E-02
NT5E	1.08	2.10E-21	6.12E-18
HEY1	1.09	8.89E-17	1.20E-13
AC124947.1	1.09	1.28E-03	4.80E-02
SLC44A5	1.09	1.29E-05	1.40E-03
LINC00973	1.09	3.43E-04	1.89E-02
COL12A1	1.11	6.37E-07	1.17E-04
AZIN1-AS1	1.12	8.84E-04	3.72E-02
AC004057.1	1.12	2.17E-15	2.37E-12
ITIH6	1.12	2.77E-15	2.94E-12
TGFA	1.12	7.72E-20	1.69E-16
VPS28	1.13	3.96E-04	2.11E-02
ZEB1-AS1	1.13	4.03E-04	2.13E-02
SLCO4A1-AS1	1.14	1.09E-05	1.21E-03
CXCL8	1.15	2.40E-06	3.69E-04
ITPK1	1.15	1.22E-06	2.06E-04
AC090673.1	1.16	1.25E-03	4.75E-02
ZYX	1.16	3.72E-04	2.01E-02
LINC01389	1.17	3.55E-04	1.93E-02
BMP2	1.17	6.65E-04	3.07E-02
FP325330.3	1.18	8.87E-04	3.73E-02
AC091173.1	1.18	9.65E-05	7.07E-03
RAD17	1.18	1.08E-05	1.21E-03
TJP1	1.19	6.13E-04	2.92E-02
PAPPA	1.19	2.93E-04	1.66E-02
AHNAK2	1.21	8.06E-08	1.93E-05
TUBA4A	1.21	1.08E-04	7.76E-03
AK4P3	1.21	5.57E-04	2.69E-02
CEP170	1.21	9.54E-09	2.91E-06
VIT	1.22	1.05E-11	5.83E-09
MYEOV	1.22	1.77E-04	1.14E-02
LGI3	1.27	4.50E-04	2.29E-02
RPL41P1	1.27	1.16E-07	2.70E-05

Table S2A. List of genes differentially expressed in the NME1^{LOW} subpopulation (continued).

Gene	Log Fold Change	P-Value	False Discovery Rate
RYR2	1.28	2.81E-04	1.61E-02
SMIM10L2A	1.28	2.04E-04	1.25E-02
HIST1H4H	1.29	3.13E-05	2.89E-03
ABCA10	1.29	7.14E-04	3.20E-02
CHORDC1	1.29	2.73E-08	7.52E-06
PCDHGA4	1.30	3.56E-09	1.21E-06
AC012085.1	1.33	1.13E-08	3.34E-06
AC215219.1	1.35	1.79E-04	1.15E-02
LINC01762	1.35	8.23E-04	3.52E-02
SLCO4A1	1.35	3.24E-13	2.46E-10
AL121772.1	1.37	2.60E-06	3.91E-04
CD70	1.37	3.09E-04	1.74E-02
NTRK2	1.37	1.05E-06	1.81E-04
PDE2A	1.42	9.75E-04	4.01E-02
NPTX1	1.42	2.13E-04	1.30E-02
FAM20C	1.42	8.98E-19	1.65E-15
PHRF1	1.42	6.22E-04	2.96E-02
MIR100HG	1.42	2.77E-08	7.59E-06
KIAA1456	1.43	1.50E-04	9.99E-03
FAM230C	1.44	3.81E-05	3.36E-03
CLCA2	1.44	4.11E-04	2.15E-02
LYPD1	1.44	7.38E-05	5.77E-03
FYB	1.47	1.59E-05	1.67E-03
NAPEPLD	1.48	9.57E-06	1.09E-03
CLDN4	1.48	9.11E-06	1.04E-03
RAMP1	1.49	1.16E-18	2.03E-15
PLPP4	1.49	4.52E-08	1.15E-05
AC002384.2	1.51	5.84E-07	1.09E-04
RPL21P93	1.52	1.38E-09	5.20E-07
GFRA1	1.53	1.70E-08	4.91E-06
MGLL	1.53	1.72E-17	2.51E-14
PPP1R14C	1.61	1.84E-12	1.19E-09
SCARA5	1.62	2.80E-14	2.33E-11
YTHDC1	1.62	5.72E-05	4.72E-03
AREG	1.63	1.35E-04	9.28E-03
CDH13	1.63	4.11E-13	2.94E-10
MPP4	1.65	1.40E-13	1.12E-10
SLC14A1	1.66	6.44E-06	7.83E-04
IGFBP5	1.67	2.08E-10	9.01E-08
AKR1C1	1.71	7.23E-09	2.24E-06
BHLHE41	1.74	1.69E-06	2.69E-04
RASAL1	1.85	1.22E-06	2.06E-04

Table S2A. List of genes differentially expressed in the NME1^{LOW} subpopulation (continued).

Gene	Log Fold Change	P-Value	False Discovery Rate
TMEM50B	1.88	7.95E-05	6.06E-03
CACNA2D4	1.88	4.70E-09	1.52E-06
CCDC81	1.88	2.04E-04	1.25E-02
AL158064.1	1.94	7.09E-04	3.18E-02
DCAF11	1.96	1.86E-09	6.70E-07
CCDC162P	1.96	7.47E-04	3.29E-02
AL133284.1	1.98	5.03E-04	2.49E-02
EPHA4	1.99	8.84E-06	1.03E-03
PTEN	2.06	5.28E-16	6.38E-13
CU634019.1	2.07	1.09E-03	4.34E-02
DUS4L	2.08	1.62E-05	1.69E-03
AC156455.1	2.10	2.33E-05	2.31E-03
THSD4	2.10	2.72E-09	9.42E-07
PSG4	2.12	7.48E-05	5.81E-03
CU638689.4	2.15	5.09E-05	4.30E-03
AKT3	2.18	8.15E-13	5.60E-10
PCSK1	2.18	6.31E-10	2.48E-07
SCN4B	2.19	1.27E-03	4.78E-02
APCDD1L	2.21	4.81E-04	2.42E-02
HEPHL1	2.27	1.39E-04	9.46E-03
AC092368.3	2.31	6.26E-04	2.96E-02
KLF13	2.31	1.44E-05	1.53E-03
AL161431.1	2.32	7.55E-04	3.32E-02
LINC00462	2.36	8.82E-04	3.71E-02
AC002056.1	2.50	1.30E-03	4.89E-02
PINX1	2.57	5.20E-07	9.90E-05
RPL4P5	2.58	1.00E-04	7.28E-03
DHRS4	2.70	1.44E-08	4.23E-06
SPATC1L	2.77	3.73E-07	7.25E-05
NPIPA8	2.80	1.62E-04	1.05E-02
NUDT4P1	2.88	2.66E-16	3.45E-13
SLC14A2	3.15	4.86E-04	2.43E-02
TRIML2	3.15	3.65E-08	9.48E-06
PARN	3.42	5.91E-32	4.14E-28
AP000561.1	3.44	3.58E-06	5.11E-04
IGKV1OR-3	3.65	1.01E-03	4.11E-02
SERTAD4	4.18	5.04E-12	2.94E-09
ACTA2-AS1	4.49	8.92E-04	3.74E-02
MIR3648-2	4.98	7.62E-05	5.91E-03
PEG13	5.12	3.07E-05	2.85E-03
SPINK1	5.29	7.71E-06	9.22E-04
RPS18	5.66	2.57E-136	8.98E-132

Table S2A. List of genes differentially expressed in the NME1^{LOW} subpopulation (continued).

Gene	Log Fold Change	P-Value	False Discovery Rate
RHEBP1	6.86	2.16E-23	8.39E-20
PPP3R1	-1.01	2.17E-07	4.69E-05
CDKN1B	-1.01	1.21E-07	2.79E-05
NAGS	-1.01	1.10E-03	4.34E-02
IPO7	-1.03	4.14E-19	8.06E-16
P2RX5	-1.03	4.92E-06	6.36E-04
APP	-1.03	9.60E-25	4.80E-21
FKBP10	-1.03	4.61E-21	1.24E-17
CBX1	-1.04	1.83E-14	1.69E-11
GET4	-1.04	4.19E-05	3.65E-03
AGAP2-AS1	-1.05	6.55E-04	3.04E-02
UBBP4	-1.07	1.09E-06	1.87E-04
LIMS2	-1.09	1.02E-03	4.15E-02
AL121594.1	-1.11	8.67E-05	6.50E-03
C15orf54	-1.11	8.08E-05	6.15E-03
EMC6	-1.12	6.27E-04	2.96E-02
GAPDHP40	-1.14	5.98E-05	4.88E-03
MAGEE1	-1.15	6.42E-04	3.01E-02
RAB11FIP4	-1.15	1.33E-06	2.18E-04
ITGA2B	-1.18	8.13E-06	9.64E-04
TGFBR3L	-1.18	4.19E-04	2.17E-02
HS6ST1P1	-1.18	1.50E-04	9.99E-03
NME2	-1.18	4.84E-05	4.13E-03
OXGR1	-1.18	2.58E-04	1.50E-02
TUBBP6	-1.18	5.30E-04	2.58E-02
SPRY3	-1.18	3.39E-04	1.87E-02
GSG2	-1.19	4.31E-09	1.42E-06
BX322650.1	-1.19	5.77E-04	2.77E-02
AC114947.2	-1.20	4.75E-04	2.39E-02
RPL30P4	-1.21	3.85E-04	2.07E-02
ADAMTS15	-1.22	3.85E-13	2.81E-10
C1QL4	-1.22	1.23E-04	8.66E-03
CCDC184	-1.24	4.09E-04	2.14E-02
IFI27L2	-1.25	2.48E-05	2.43E-03
HMGCS1	-1.27	2.46E-34	2.15E-30
ARHGEF25	-1.27	1.03E-03	4.16E-02
RANP6	-1.28	1.93E-05	1.96E-03
NPM3	-1.28	4.84E-11	2.45E-08
GNG10	-1.28	7.75E-04	3.38E-02
TNRC6C-AS1	-1.29	3.83E-04	2.06E-02
AC091133.5	-1.33	5.97E-06	7.36E-04
AL136454.1	-1.33	4.91E-04	2.45E-02

Table S2A. List of genes differentially expressed in the NME1^{LOW} subpopulation (continued).

Gene	Log Fold Change	P-Value	False Discovery Rate
NME1	-1.34	6.33E-13	4.43E-10
RAB5C	-1.35	1.49E-20	3.73E-17
AC099850.3	-1.36	4.20E-04	2.17E-02
NCCRP1	-1.36	3.35E-04	1.86E-02
IBTK	-1.37	3.27E-06	4.73E-04
ZNF114	-1.38	7.06E-07	1.28E-04
PCSK9	-1.39	4.54E-06	6.09E-04
CCDC71	-1.42	2.86E-06	4.26E-04
HIST1H1E	-1.43	2.66E-22	8.47E-19
AL590762.3	-1.53	7.79E-04	3.38E-02
PYCARD	-1.54	2.96E-05	2.77E-03
AP001922.4	-1.56	3.92E-05	3.44E-03
ELFN1	-1.59	1.83E-05	1.88E-03
RANGRF	-1.64	4.32E-06	5.86E-04
PDZD4	-1.68	7.39E-08	1.81E-05
RAD23B	-1.68	3.34E-35	3.90E-31
CORO6	-1.70	7.13E-10	2.77E-07
AP000662.1	-1.70	2.52E-05	2.45E-03
RNF31	-1.72	8.21E-06	9.71E-04
AC007066.1	-1.72	1.03E-04	7.43E-03
EXOC3L2	-1.73	1.26E-04	8.82E-03
AC113410.1	-1.77	8.29E-04	3.53E-02
MT-TY	-1.77	2.92E-06	4.32E-04
MT-TE	-1.78	8.59E-04	3.62E-02
AC009362.1	-1.79	1.19E-05	1.31E-03
TOX	-1.79	2.10E-04	1.28E-02
HIC1	-1.81	3.60E-08	9.41E-06
MPZ	-1.81	8.26E-04	3.52E-02
AP001024.1	-1.85	1.20E-03	4.59E-02
THY1	-1.89	9.17E-09	2.82E-06
SLC16A5	-1.92	9.18E-05	6.75E-03
FOPNL	-1.93	2.53E-10	1.06E-07
COL1A1	-1.98	4.28E-05	3.70E-03
F8A2	-2.01	1.41E-04	9.54E-03
TNFAIP2	-2.03	1.60E-04	1.05E-02
IGF2BP1	-2.06	2.16E-22	7.57E-19
SLFN11	-2.12	4.25E-04	2.19E-02
RPL23AP18	-2.12	1.82E-04	1.16E-02
MDFI	-2.16	1.10E-04	7.85E-03
ACTG1P9	-2.20	4.09E-04	2.14E-02
AC092670.1	-2.25	1.19E-07	2.77E-05
CD79B	-2.27	1.40E-04	9.49E-03

Table S2A. List of genes differentially expressed in the NME1^{LOW} subpopulation (continued).

Gene	Log Fold Change	P-Value	False Discovery Rate
RPL12P35	-2.32	5.04E-04	2.49E-02
ERI1	-2.37	2.74E-35	3.90E-31
AKAP5	-2.65	6.72E-04	3.09E-02
UBBP1	-2.66	1.14E-03	4.44E-02
AC009414.2	-2.68	6.83E-04	3.13E-02
AC091839.1	-2.73	2.12E-06	3.31E-04
S100A11P1	-2.77	8.99E-07	1.60E-04
NTS	-2.78	7.45E-04	3.29E-02
AC087190.1	-2.91	4.05E-10	1.65E-07
TTC16	-3.04	3.70E-07	7.24E-05
RELN	-3.28	1.50E-04	9.99E-03
AICDA	-3.54	2.34E-04	1.39E-02
RPS26P6	-3.84	1.40E-25	8.19E-22
NXPH3	-4.23	3.00E-06	4.41E-04
ZNF780B	-4.53	1.49E-24	6.51E-21
SCUBE1	-4.63	2.31E-04	1.38E-02
CT45A2	-4.68	6.25E-04	2.96E-02
AC090543.2	-4.68	3.78E-17	5.29E-14
AC138517.2	-4.97	1.26E-04	8.82E-03

Table S2B. The NME1^{LOW} subpopulation exhibits elevated expression of genes related to two GO Biological Processes.

GO Biological Process	Number of Genes	Expected Number of Genes	Fold Enrichment	P-Value	False Discovery Rate
Anatomical Structure Morphogenesis	38	9.7	3.92	2.45E-06	3.82E-02
Tube Development	15	3.8	3.95	6.27E-06	4.93E-02

Table S2C. Biological processes related to anatomical structure/morphogenesis that are altered in the NME1^{LOW} subpopulation.

Process	Fold Enrichment	P-Value	False Discovery Rate	Number of Genes	Genes
Angiogenesis	17.89	1.01E-07	4.36E-05	7	CXCL8, HEY1, SLIT2, RAMP1, PTEN, TGFA, CDH13
Bone Development	16.49	1.02E-04	1.04E-02	4	COL12A1, FAM20C, BMP2, VIT
Positive Regulation of Neuron Differentiation	15.68	2.41E-07	7.86E-05	7	NTRK2, PLXNA2, EPHA4, SLIT2, RASAL1, PTEN, BMP2, AL133284
Negative Regulation of Cell Adhesion	12.32	3.04E-04	2.34E-02	4	PLXNA2, PTEN, CDH13, BMP2
Positive Regulation of Cell Migration	10.32	2.08E-05	2.85E-03	6	CXCL8, AKT3, IGFBP5, CDH13, BMP2, AL133284
Transmembrane Receptor Protein Tyrosine Kinase Signaling Pathway	9.30	3.71E-05	4.60E-03	6	NTRK2, AC092368, GFRA1, EPHA4, AREG, PTEN, TGFA
Heart Development	9.28	3.75E-05	4.61E-03	6	HEY1, SLIT2, PTEN, AC004057, BMP2, RYR2
Embryo Development	7.69	1.29E-06	2.80E-04	10	BHLHE41, CXCL8, ITPK1, PLXNA2, COL12A1, HEY1, TJP1, BMP2, AC090673, RYR2
Response to Growth Factor	7.49	4.83E-04	3.28E-02	8	NTRK2, AC0902368, CXCL8, ZYX, PTEN, PDE2A, BMP2, RYR2
Negative Regulation of Cell Cycle	7.34	5.31E-04	3.50E-02	8	AC012085, CXCL8, PINX1, AL161431, PTEN, BMP2, AC090673, RAD17
Regulation of Signaling Receptor Activity	7.07	6.27E-04	3.83E-02	9	CXCL8, NPTX1, LYPD1, AREG, CD70, GPRC5A, PTEN, TGFA, BMP2
Negative Regulation of Apoptotic Process	5.12	9.17E-04	4.96E-02	6	NTRK2, EPHA4, PTEN, TGFA, AC090673, AL133284,
Animal Organ Development	5.07	6.55E-12	8.53E-09	22	NTRK2, BHLHE41, CXCL8, AKT3, IGFBP5, AL161431, PLXNA2, COL12A1, HEY1, EPHA4, CLDN4, SLIT2, AREG, PTEN, PDE2A, TGFA, FAM20C, AC004057, BMP2, AC090673, RYR2, VIT

Table S2D. Individual anatomical structure/morphogenesis genes that are altered in the NME1^{LOW} subpopulation.

Anatomical Structure Morphogenesis		
Gene	Log Fold Change	False Discovery Rate
SLIT2	1.01	4.51E-02
GPRC5A	1.01	4.51E-02
PLXNA2	1.07	6.80E-04
HEY1	1.08	1.20E+13
COL12A1	1.11	1.10E-04
AC004057	1.12	2.37E-12
TGFA	1.12	1.69E-16
CXCL8	1.15	3.00E-04
ITPK1	1.15	2.00E-04
AC090673	1.16	4.70E-02
ZYX	1.16	2.01E-02
BMP2	1.17	3.07E-02
RAD17	1.18	1.20E-03
TJP1	1.19	2.92E-02
VIT	1.22	5.83E-09
RYR2	1.28	1.60E-02
AC012085	1.33	3.34E-06
CD70	1.37	1.70E-02
NTRK2	1.37	1.81E-04
PDE2A	1.42	4.01E-02
FAM20C	1.42	1.65E-15
NPTX1	1.42	1.29E-02
LYPD1	1.44	5.70E-03
CLDN4	1.48	1.00E-03
RAMP1	1.49	2.03E-15
GFRA1	1.53	4.91E-06
AREG	1.63	9.00E-04
CDH13	1.63	2.94E-10
IGFBP5	1.67	9.01E-08
BHLHE41	1.74	2.60E-04
RASAL1	1.85	2.00E-04
AL133284	1.98	2.40E-02
EPHA4	1.99	1.03E-03
PTEN	2.06	6.38E-13
AKT3	2.18	5.60E-10
AC092368	2.31	2.90E-02
AL161431	2.32	3.32E-02
PINX1	2.57	9.90E-05

Table S2E. Individual tube development genes that are altered in the NME1^{LOW} subpopulation.

Tube Development		
**Gene**	**Log Fold Change**	**False Discovery Rate**
SLIT2	1.01	4.51E-02
PLXNA2	1.07	6.80E-04
HEY1	1.08	1.20E+13
TGFA	1.12	1.69E-16
CXCL8	1.15	3.00E-04
ITPK1	1.15	2.00E-04
BMP2	1.17	3.07E-02
RYR2	1.28	1.60E-02
NTRK2	1.37	1.81E-04
RAMP1	1.49	2.03E-15
AREG	1.63	9.00E-04
CDH13	1.63	2.94E-10
IGFBP5	1.67	9.01E-08
EPHA4	1.99	1.03E-03
PTEN	2.06	6.38E-13

Table S2F. The GO Cellular Component "Receptor Complex" is upregulated in the NME1^{LOW} subpopulation.

GO Cellular Component	Number of Genes	Expected Number of Genes	Fold Enrichment	P-Value	False Discovery Rate
Receptor Complex	6	0.51	11.78	9.94E-06	1.98E-02

Table S2G. Individual "Receptor Complex" genes that are altered in the NME1^{LOW} subpopulation.

Receptor Complex		
**Gene**	**Log Fold Change**	**False Discovery Rate**
PLXNA2	1.07	6.88E-04
BMP2	1.17	3.07E-02
NTRK2	1.37	1.89E-04
RAMP1	1.49	2.03E-15
GFRA1	1.53	4.91E-06
EPHA4	1.99	1.03E-03

Table S3. Expression of stem cell genes that are not altered in the NME1^{LOW} subpopulation.

Gene	Log Fold Change	False Discovery Rate
ABCG2	-0.54	0.192
CCNB1	-0.16	0.839
JARID1	0.03	1.000
PROM1 (CD133)	0.03	1.000
SALL4	0.13	1.000
MLANA	0.13	1.000
CD44	0.16	0.664
ABCB5	0.16	1.000
SOX10	0.20	0.462
SOX2	0.32	0.336
ALDH1	0.42	0.520
KLF4	0.58	0.116
ABCB1	0.65	0.707
CD34	0.88	0.670
CXCR6	1.07	0.732
NANOG	below detection	N.A.
OCT4	below detection	N.A.

Table S4. Oligodeoxynucleotide primers used for qRT-PCR measurements of relevant transcripts.

Gene	Orientation	Sequence (5'-3')
AKT3	For	ATGCTGGACAAAGATGGC
	Rev	CAGAATGTCTTCATGGTG
GFRA1	For	AAGGAGACCAACTTCAGC
	Rev	CTTCTCCTTCTTCATACC
NME1	For	CTGAATGTGGTGAAGACGG
	Rev	TATGCAGAAGTCTCCACGG
SLC14a2	For	TTCCATCTTCAGCAAG
	Rev	AAGAAGAGGTTGTAGT
